# SP1 regulates BMSC osteogenic differentiation through the miR-133a-3p/MAPK3 axis: SP1 regulates osteogenic differentiation of BMSCs

**DOI:** 10.1186/s13018-024-04889-4

**Published:** 2024-07-09

**Authors:** Liying Zhong, Yehai Sun, Cong Wang, Runzhi Liu, Wenjuan Ru, Wei Dai, Ting Xiong, Aimin Zhong, Shundong Li

**Affiliations:** Department of Geriatrics, The Third Hospital of Changsha, No. 176 Laodongxi Road, Tianxin District, Changsha, Hunan Province 410015 China

**Keywords:** SP1, MiR-133a-3p, MAPK3, Osteoporosis, BMSCs

## Abstract

**Background:**

The progression of osteoporosis (OP) can dramatically increase the risk of fractures, which seriously disturb the life of elderly individuals. Specific protein 1 (SP1) is involved in OP progression. However, the mechanism by which SP1 regulates OP progression remains unclear.

**Objective:**

This study investigated the mechanism underlying the function of SP1 in OP.

**Methods:**

SAMP6 mice were used to establish an in vivo model of age-dependent OP, and BALB/c mice were used as controls. BMSCs were extracted from two subtypes of mice. Hematoxylin and eosin staining were performed to mark the intramedullary trabecular bone structure to evaluate histological changes. ChIP assay was used to assess the targeted regulation between SP1 and miR-133a-3p. The binding sites between MAPK3 and miR-133a-3p were verified using a dual-luciferase reporter assay. The mRNA levels of miR-133a-3p and MAPK3 were detected using quantitative reverse transcription polymerase chain reaction (RT-qPCR). The protein expression of SP1, MAPK3, Colla1, OCN, and Runx2 was examined using Western blotting. Alkaline phosphatase (ALP) kit and Alizarin Red S staining were used to investigate ALP activity and mineralized nodules, respectively.

**Results:**

The levels of SP1 and miR-133a-3p were upregulated, whereas the expression of MAPK3 was downregulated in BMSCs from SAMP6 mice, and miR-133a-3p inhibitor accelerated osteogenic differentiation in BMSCs. SP1 directly targeted miR-133a-3p, and MAPK3 was the downstream mRNA of miR-133a-3p. Mechanically, SP1 accelerated osteogenic differentiation in BMSCs via transcriptional mediation of the miR-133a-3p/MAPK3 axis.

**Conclusion:**

SP1 regulates osteogenic differentiation by mediating the miR-133a-3p/MAPK3 axis, which would shed new light on strategies for treating senile OP.

**Supplementary Information:**

The online version contains supplementary material available at 10.1186/s13018-024-04889-4.

## Introduction

Osteoporosis (OP) is a frequent bone disease among elderly individuals characterized by bone formation imbalance [1] and can increase fracture risk and decrease bone strength [2, 3]. Currently, the prevalence of OP is increasing and disturbing the health of humans [4–6]. Dysfunction of bone marrow mesenchymal stem cells (BMSCs) is associated with the pathogenesis of OP, which induces various metabolic diseases [7–9]. Hence, studying osteogenesis in BMSCs for treating OP is of great significance.

Specific protein 1 (SP1) is a transcription factor mediated by SUMOylation modifiers, which is upregulated in tumors; moreover, it often acts as a key regulator in inflammatory responses [10, 11]. Studies have shown that SP1 is an activated transcription factor in primary OP, and its locus genotyping plays a vital role in OP [12]. However, the mechanism by which SP1 participates in OP progression remains unexplored. Noncoding RNAs have been reported to act as key modulators in bone diseases [1, 13]. Furthermore, miRNAs are noncoding RNA sequences with approximately 18–22 base pairs in length [13], which can regulate the progression of bone diseases (including OP) [14]. Dysregulation of miRNAs can lead to the occurrence of multiple diseases (e.g., malignant tumors and inflammation) [15, 16]; more importantly, miRNAs are reported to participate in the physiopathology, diagnosis, and therapeutic challenge of OP [14]. As an important component of epigenetic modification, miRNAs are involved in ontogeny and bone and joint diseases. For instance, Ding et al. demonstrated that the upregulation of miR-224-5p inhibited OP progression by regulating Runx2 and Sp7 [17]; miR-155-5p reversed circ_0114581-induced osteogenic differentiation in BMSCs, which further attenuated the development of OP [18]. As a highly conserved miRNA that functions in skeletal and cardiac muscles, miR-133a-3p is a crucial modulator of bone metabolism. Peng et al. reported that miR-133a-3p inhibited fracture healing by modulating the RUNX2/BMP2 axis [19]. Moreover, miR-133a-3p is involved in the osteogenic differentiation of vascular smooth muscle cells [20]. Interestingly, miR-133a-3p is increased in BMSCs and inhibits osteogenic differentiation in postmenopausal patients with OP [21]. Meanwhile, the analysis of JASPAR (http://jaspar.genereg.net/) showed that SP1 had binding sites with the miR-133a-3p promoter region. However, the detailed relationship between SP1 and miR-133a-3p in osteogenic differentiation during OP progression remains largely unknown.

Mitogen-activated protein kinase 3 (MAPK3) is activated by multiple stimuli [22]. MAPK3 and related downstream signaling pathways have been shown to be important regulators of OP progression [23, 24]. Wang et al. reported that Novel Soy Peptide CBP promotes osteoblast differentiation in OP through activation of the MAPK pathway [25]. These findings indicated that MAPK3-derived osteogenic differentiation is associated with the occurrence of OP. We initially identified potential binding sites between MAPK3 and miR-133a-3p by biological information analysis, indicating that miR-133a-3p participates in osteogenic differentiation in OP by mediating MAPK3.

In this study, we hypothesized that SP1 regulates osteogenic differentiation in BMSCs through mediation of the miR-133a-3p/MAPK3 axis. This study would provide a theoretical basis for exploring new strategies for managing OP.

## Materials and methods

### In vivo study and BMSC isolation

Four-month-old male SAMP6 and BALB/c mice were purchased from Casgene Biotech (210–250 g, China). The protocol was approved by the Ethics Committee of The Third Hospital of Changsha. After being anesthetized with isoflurane, the mice were euthanized after being treated with carbon dioxide for 10 min. Next, as described above [26], the femur and tibia of the mice were immediately removed for the isolation of BMSCs. BMSCs were rinsed using a needle system and cultured in Dulbecco’s Modified Eagle Medium (DMEM) (Invitrogen, Shanghai, China) containing fetal bovine serum (FBS) (10%, Solarbio). After the first passage, osteoblast differentiation was induced for 21 days with osteogenic differentiation induction medium (OM), which consisted of high glucose DMEM containing 10% FBS, 100 IU/ml penicillin/streptomycin, 100 nM dexamethasone, 0.2 mM ascorbic acid, and 10 mM β-glycerophosphate (Sigma, St. Lious, MA, USA). Finally, the morphology of these cells was observed using a microscope. Cells were identified using flow cytometry. In contrast, to detect the function of SP1 in OP, SAMP6 mice were administered SP1 lentivirus shRNA (sh-SP1, 200 µL, GenePharma, Shanghai, China) and corresponding negative control (sh-NC, GenePharma) via the tail vein three times a week. At the end of this study, the mice were euthanized for collecting serum and femoral tissues.

### Cell transfection

MiR-133a-3p mimics/inhibitor and the corresponding negative control were obtained from Sangon Biotech (Shanghai, China). BMSCs were transfected with the aforementioned plasmids using Lipofectamine™ 3,000 (Invitrogen, Shanghai, China) according to the manufacturer’s instructions. Meanwhile, full-length SP1 and MAPK3 cDNA were cloned using a PCMV vector (Fenghbio, Changsha, China). Stable transfection was performed using the pLV-cDNA lentiviral-mediated expression system (Fenghbio), and lentiviral DNA was then packaged. The lentiviral-containing medium was filtered and transferred to BMSCs after transfection. The infected cells were treated with puromycin (1 µg/mL, Sigma, St. Louis, MA, USA) for a week. Stable expression clones were used in subsequent analyses.

### Hematoxylin and eosin (H&E) staining

An in vivo model of OP was established using 4-month-old male SAMP6 mice, and BALB/c mice were used as controls. The experiments were performed according to the animal protection and use guidelines of The Third Hospital of Changsha. In detail, paraffin sections were melted and dewaxed for 10 min (2 rounds). Then, the paraffin sections were hydrated with gradient ethanol series (100%, 90%, 80%, and 70% ethanol), and the sections were then immersed for 5 min after washing. After that, the sections were rinsed and underwent acidic differentiation. Eosin staining was performed. Finally, the sections were dehydrated with ascending ethanol solutions before sealing. H&E staining (Beyotime) was performed to detect mouse femur bone slices, as previously reported [27].

### Procollagen type I N-terminal propeptide (PINP) detection

The concentration of PINP in the SAMP6 mice serum or BMSCs was detected using commercial kits (cat.no H285, Jiancheng Co. Ltd, Nanjing, China) according to the manufacturer’s protocol. The related procedure was performed as previously described [28].

### Immunohistochemical (IHC) staining

Paraffin-embedded mouse femur bone slices were dewaxed, rehydrated, and incubated in H_2_O_2_ (0.3%) to eliminate endogenous peroxidase activity. Antigen repair was soaked for 30 min. SP1 and MAPK3 antibodies (Abcam, Shanghai, China) were incubated with mouse femur bone slices at 4 °C for 12 h, and the poly-horse radish peroxidase anti-rabbit immunoglobulin detection system (Abcam) was used with a PV6001 two-step method (Zhongshanjinqiao). As the operating protocol, chromogenic reaction was performed with 3, 3-diaminobenzidine (Solarbio, DA1015), and hematoxylin (Solarbio, G1080) was used as the counterstain. Finally, images were captured using a Nikon microscope.

### Quantitative reverse transcription polymerase chain reaction (RT-qPCR)

TRIzol reagent (Invitrogen) was used to extract total RNA. A reverse transcription kit (TaKaRa, Ver.3.0) was used to synthesize cDNA. RT-qPCRs were performed as follows: 94 °C for 2 min, followed by 35 cycles of 94 °C for 30 s and 55 °C for 45 s. The sequences were designed and obtained by GenePharma, as follows: MAPK3: F, 5′-CTATGCCTCTGGACGCACAAC-3′ and R, 5′-CCCATCAGGCAACTCGTAACTC-3′; GAPDH: F, 5′-CTACCCCATCCGTATCTGGCT-3′ and R, 5′-GGTCTGTGTCGCGCCCTGCCA-3′; miR-133a-3p: F, 5′- AATCGAATTTTCTCGCATCT-3′ and R 5′-TCAATGCCCAAAAATGCCCGC-3′. U6: F, 5′-ACTCGTCCACTTCACGAC-3′ and R 5′- GCTTACCGATCGCCCATCTT-3′. Relative fold changes were calculated using the 2^−ΔΔCt^ method. U6 or glyceraldehyde 3-phosphate dehydrogenase was used for normalization. The RT-qPCR procedure was performed as previously described [29].

### ChIP

The ChIP assay was performed according to the operating protocol of the EZ-ChIPTM kit. Specific antibodies or negative control antibodies were used to immunoprecipitate chromatin fragments (200–500 bp). Protein–DNA complex-released DNA was purified using magnetic beads for RT-qPCR analysis. In brief, cells were treated with Tan 2 A (20 µM) for 16 h at 37 °C. Furthermore, 275 µL 37% formaldehyde (Sangon Biotech Co., Ltd.) was added to 10 mL of growth media to cross-link protein to chromatin for 10 min at room temperature and then quenched with glycine at a final concentration of 125 mM immediately at room temperature for 5 min. Cells were harvested in cold phosphate-buffered saline. The cellular nuclear pellets were resuspended in sonication buffer. The resulting cell suspension was sheared by sonication (MiSonix Sonicator 3,000) on ice using 30/30 s on/off for 10 min (Output Power: 4). ChIP assay was performed using 1.5 µg rabbit IgG (1.15 mg/ml, ProteinTech Group, 30000-0-AP, Shanghai, China) or anti-HIF-1α antibodies (CST, #14,179, Shanghai, China) bound to the magnetic protein G beads. Each IP required the addition of 500 µL dilution buffer. The protein G bead–antibody/chromatin complex was washed by resuspending the beads in 0.5 mL of each of the cold buffers in the following order: Low Salt Immune Complex Wash Buffer, High Salt Immune Complex Wash Buffer, High Salt Immune Complex Wash Buffer, and High Salt Immune Complex Wash Buffer. The DNA fragment containing the HRE site was amplified by qPCR (SYBR-Green Master Mix, Takara Biotechnology Co., Ltd.)

### Dual-luciferase reporter assay

The potential binding sites of miR-133a-3p and MAPK3 or SP1 and the miR-133a-3p promoter region were predicted using online bioinformatics prediction. In detail, the wild type (WT) or mutated type (MUT) of the MAPK3 3′-untranslated region (UTR) was constructed using a vector (pmiRGLO, GenePharma) called WT/MUT-MAPK3. Lipofectamine 2000 was applied for co-transfecting cells with WT/MUT-MAPK3 and miRNAs. After transfection, a dual-luciferase reporter system (Promega) was used to measure luciferase activity. To investigate the binding between MIR133A1 and SP1, the WT or MUT of the MIR133A1 promoter region was established based on a pmiRGLO vector (GenePharma) and named WT/MUT-MIR133A1. Lipofectamine 2000 was used in cotransfecting cells with WT/MUT-MIR133A1 and oe-SP1 or oe-NC. After transfection, a dual-luciferase reporter system (Promega) was employed to measure luciferase activity. The experiments were performed as previously described [30].

### Western blotting

Total protein was isolated from cells or tissues using RIPA (Beyotime, Shanghai, China). Proteins were separated using sodium dodecyl-sulfate polyacrylamide gel electrophoresis (10%) and then transferred onto polyvinylidene fluoride membranes (Beyotime). The membranes were blocked with skim milk (5%) for 1 h. The membranes were then incubated with specific antibodies overnight, including SP1 (Abcam, ab124804, 1:1,000), MAPK3 (Abcam, ab266519, 1:1,000), Colla1 (Cell Signaling,39,952, 1:1,000), OCN (Abcam, ab133612, 1:1,000), and Runx2 (Abcam, ab192256,1:1,000), at 4 °C. Afterward, the membranes were incubated with goat anti-rabbit IgG H&L (Abcam, ab6721, 1:2000) for 1 h, and protein bands were then detected using a gel imaging system (Bio-Rad). The density of the bands was analyzed using ImageJ (version 2.0; NIH, USA).

### Alizarin red S staining

Alizarin red S staining (Beyotime) was performed to analyze calcification after bone formation of BMSCs. BMSCs were cultured in OM for 3 weeks after washing. Formalin (10%) was used to fix BMSCs for 30 min. BMSCs were dyed for 3 h after washing. Finally, the BMSCs were cleaned with distilled water and photographed under a microscope.

### Alkaline phosphatase (ALP) activity detection

An ALP assay kit (Beyotime, Nanjing, China) was used to determine ALP activity. Radioimmunoprecipitation buffer supplemented with 1 mM SF was used to lyse BMSCs. The resulting lysate was centrifuged for 30 min at 10,000 RPM. pNPP reagent (200 µL; Bio-Rad Laboratory, California, USA) was used to incubate the supernatant for 30 min. ALP activity was normalized at 405 nm using a spectrophotometer.

### Statistical analysis

Data are expressed as means ± standard deviations, and the experiments were repeated three times in each group. Student’s *t*-test for two groups or one-way analysis of variance, followed by Tukey’s multiple comparison test, was used to compare the differences. *P-*values < 0.05 were used to denote statistical significance.

## Results

### SP1 and miR-133a-3p were upregulated and MAPK3 was downregulated during osteogenic differentiation

**Fig. 1:**
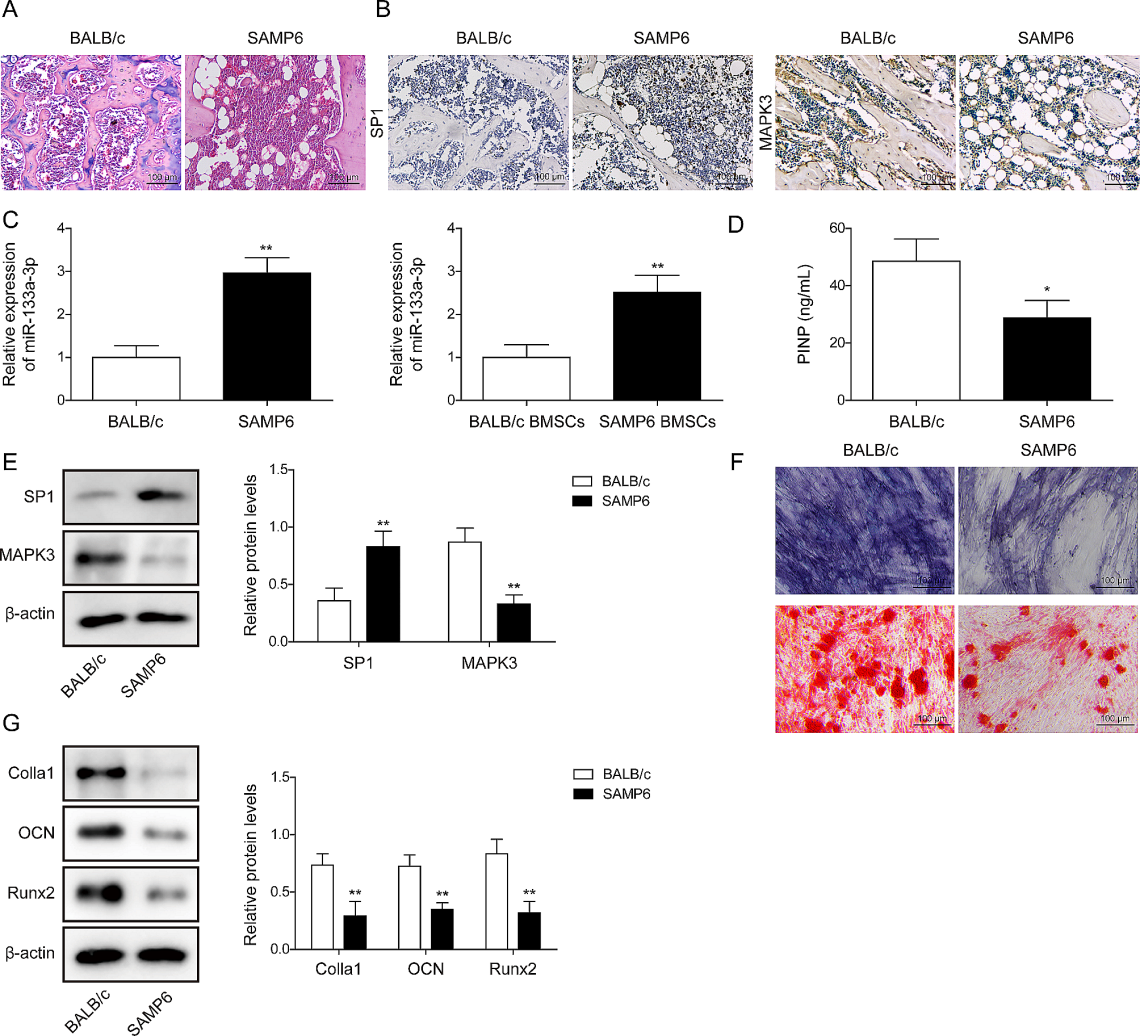
SP1 and miR-133a-3p were upregulated, MAPK3 was downregulated, and osteogenic differentiation was inhibited in SAMP6 mouse-derived BMSCs. SAMP6 mice were considered as an in vivo model of age-dependent OP, and BALB/c mice were considered as controls. **A**, The histological changes in OP were investigated using H&E staining (The scale bar was 100 μm). **B**, the levels of SP1 and MAPK3 were detected using IHC staining (the scale bar was 100 μm). **C**, the level of miR-133a-3p in mouse femur bone tissues and BMSCs was investigated using RT-qPCR. **D**, The contents of PINP in the serum of mice were assessed using commercial kits. **E**, The expression of SP1 and MAPK3 was examined using Western blotting. **F**, ALP activity was detected using the Alkaline Phosphatase Assay Kit, and Alizarin Red S staining was performed to examine the mineralized nodules after culture with osteogenic OM for 21 days (the scale bar was 100 μm). **G**, The contents of PINP in the serum of mice were assessed using commercial kits. The protein levels of Colla1, OCN, and Runx2 were assessed using Western blotting. *N* = 8, **p < 0.01

An in vivo model of age-dependent OP was established using SAMP6 mice. H&E staining was performed to evaluate mouse femur bone sections. The results revealed that the bone trabeculae were sparse and the bone mineral density decreased in the femurs of SAMP6 mice compared with those of BALB/c mice (Fig. 1A). Furthermore, the level of SP1 in SAMP6 mice was higher than that in BALB/c mice. However, the expression of MAPK3 was significantly lower in SAMP6 mice than in BALB/c mice (Fig. 1B). Subsequently, BMSCs derived from SAMP6 and BALB/c mice were cultured with OM for 3 weeks; the results demonstrated an upregulation of miR-133a-3p in SAMP6 and SAMP6 mouse-derived BMSCs (Fig. 1C). The levels of MAPK3 and PINP in SAMP6 mice were much lower than those in BALB/c mice while SP1 expression in SAMP6 mice was upregulated (Fig. 1D). Moreover, as shown in Fig. 1E, ALP activity and mineralized nodules in BMSCs separated from SAMP6 mice were lower than those in BALB/c mice. The protein levels of Colla1, OCN, and Runx2 in SAMP6 mouse-derived BMSCs were much lower (Fig. 1F). All these data indicated that an in vivo model of OP was successfully established, accompanied by regulating SP1, miR-133a-3p, and MAPK3.

### MiR-133a-3p inhibitor accelerates osteogenic differentiation in BMSCs

**Fig. 2:**
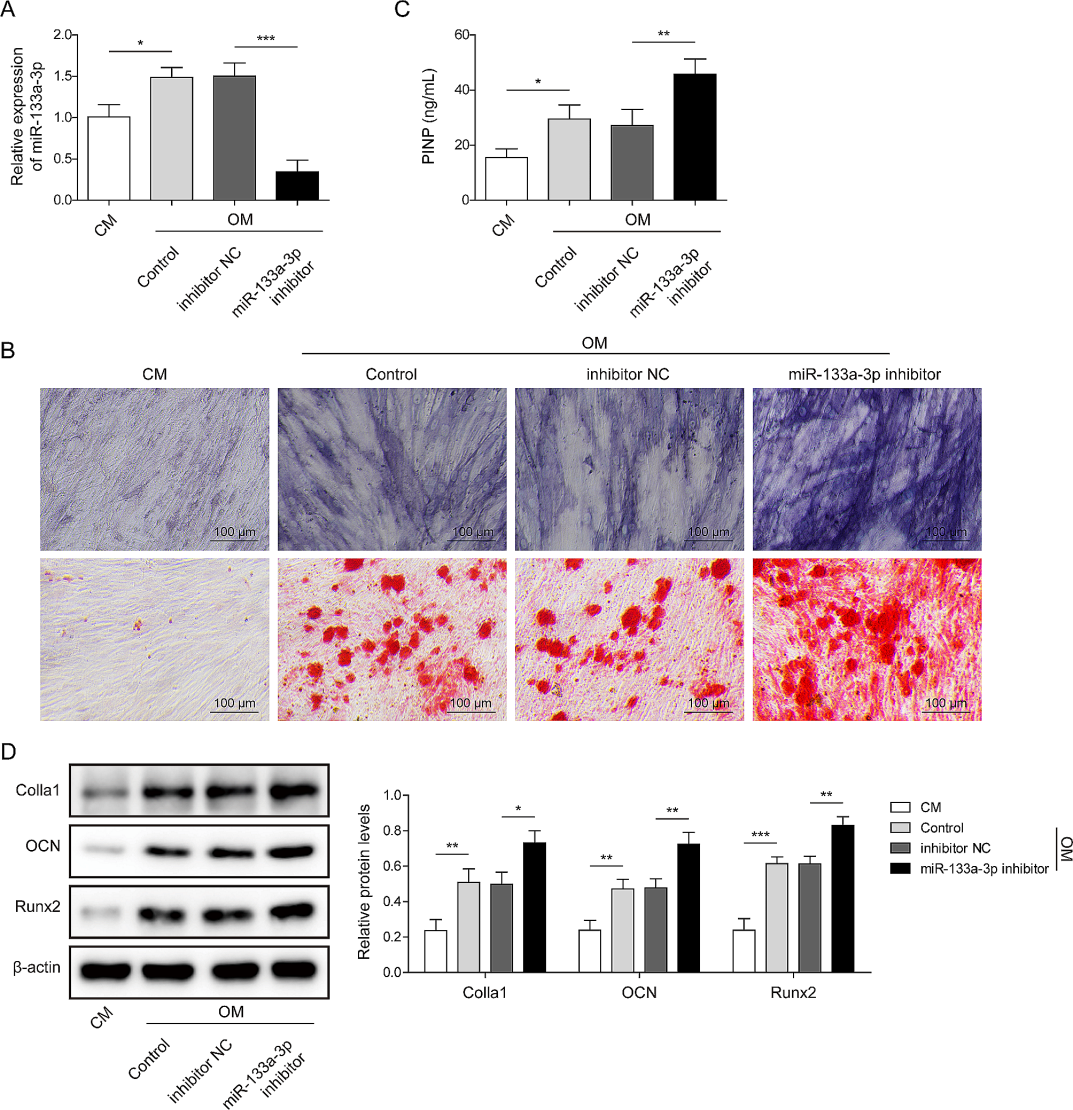
miR-133a-3p inhibitor accelerated the osteogenic differentiation of BMSCs. BMSCs were derived from the bone tissues of SAMP6 mice and then cultured with OM for 21 days. BMSCs were transfected with an miR-133a-3p/NC inhibitor. BMSCs as blank controls were cultured in CM. **A**, The level of miR-133a-3p in BMSCs was detected using RT-qPCR. **B**, Alkaline Phosphatase Kit was used to detect ALP activity, and Alizarin Red S staining was used to examine the mineralized nodules (the scale bar was 100 μm). **C**, The contents of PINP in BMSCs derived from mice were investigated using commercial kits. **D**, The protein levels of Colla1, OCN, and Runx2 in BMSCs were detected using Western blotting. *N* = 3, *p < 0.05, **p < 0.01, ***p < 0.001

To investigate the function of miR-133a-3p in the osteogenic potential of BMSCs, BMSCs were cultured in OM for 21 days and then transfected with an miR-133a-3p inhibitor. BMSCs cultured in common cell medium (CM) served as controls. The data indicated that the expression of miR-133a-3p was upregulated after osteogenic induction, which was abolished following treatment with an miR-133a-3p inhibitor (Fig. 2A). BMSCs had no osteogenic differentiation ability in the CM group. ALP activity and mineralized nodules of BMSCs were elevated after incubation with OM, and the miR-133a-3p inhibitor reversed this phenomenon (Fig. 2B). Consistently, the levels of PINP, Colla1, Runx2, and OCN were increased in BMSCs under OM, and this phenomenon was reversed by the miR-133a-3p inhibitor (Fig. 2C-D). Collectively, the downregulation of miR-133a-3p promoted osteogenic differentiation in BMSCs.

### SP1 accelerated the osteogenic differentiation of BMSCs by transcriptional miR-133a-3p modulation

**Fig. 3:**
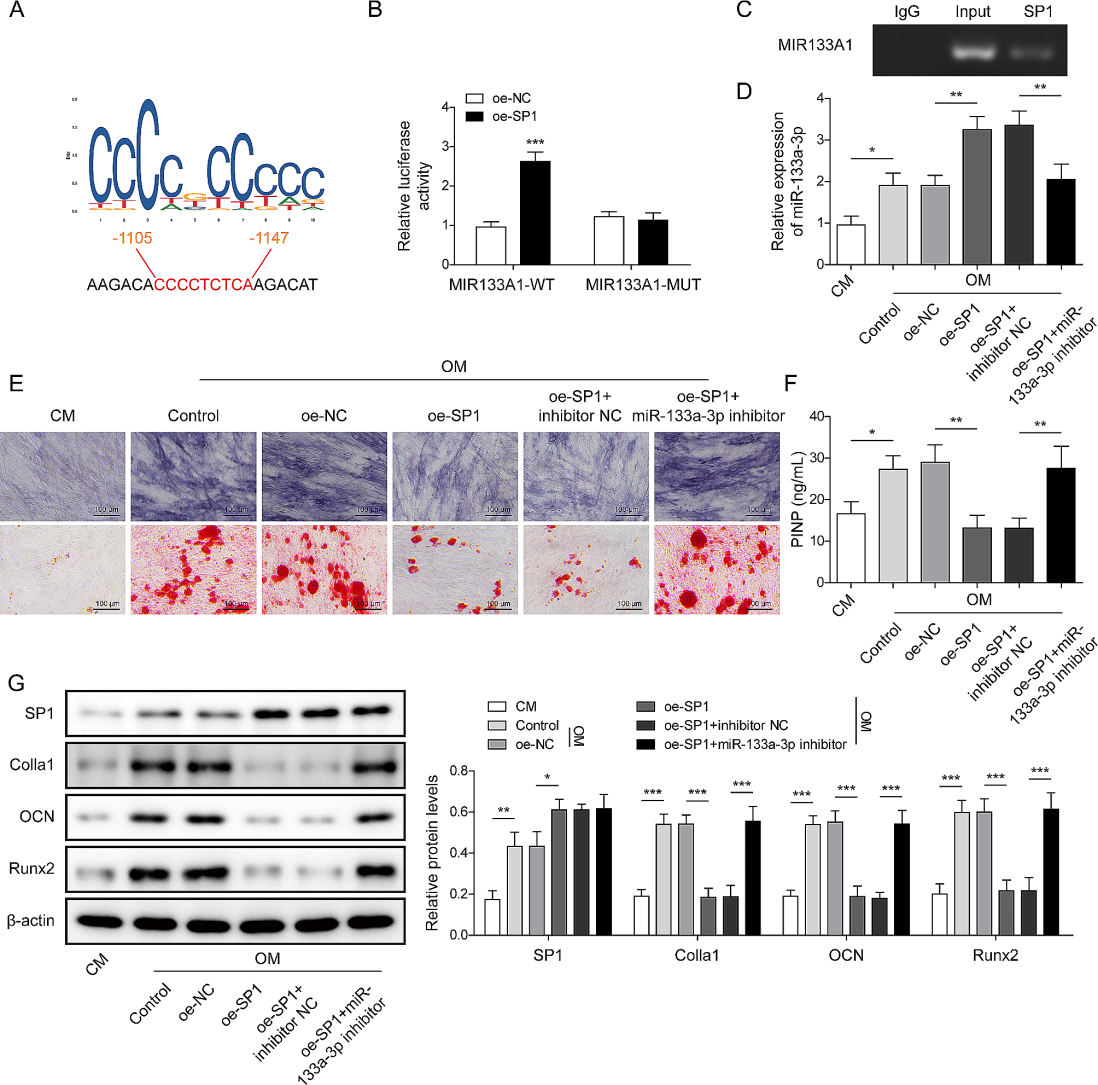
SP1 accelerated the osteogenic differentiation of BMSCs by transcriptionally modulating miR-133a-3p. **A**, The binding site of SP1 and MIR133A1 promoter region were analyzed using JASPAR. **B**, The binding relationship between SP1 and the MIR133A1 promoter region was verified using the ChIP assay. **C**, The binding between SP1 and the MIR133A1 promoter region was verified using dual-luciferase reporter assay. Subsequently, BMSCs were transfected with oe-SP1, oe-NC, oe-SP1 + NC inhibitor, or oe-SP1 + miR-133a-3p inhibitor. **D**, The level of miR-133a-3p in BMSCs was examined using RT-qPCR. **E**, ALP activity was detected using the Alkaline Phosphatase Assay Kit, and Alizarin Red S staining was performed to analyze mineralized nodules (the scale bar was 100 μm). **F**, The contents of PINP in BMSCs were examined using commercial kits. **G**, The protein expression of SP1, Colla1, OCN, and Runx2 was examined using Western blotting. *N* = 3, *p < 0.05, **p < 0.01, ***p < 0.001

To explore the potential mechanisms underlying the function of miR-133a-3p in OP, Starbase (http://starbase.sysu.edu.cn/index.php) was used. The results showed that SP1 had a binding site with the MIR133A1 promoter region (Fig. 3A) and that SP1 could directly interact with the MIR133A1 promoter (Fig. 3B). Moreover, the overexpression of SP1 increased the luciferase activity in WT-MIR133A1, whereas oe-SP1 did not affect luciferase activity in MUT-MIR133A1 (Fig. 3C). Subsequently, BMSCs were cultured in OM for 3 weeks and transfected with oe-SP1 and/or an miR-133a-3p inhibitor. As indicated in Fig. 3D, the expression of miR-133a-3p was upregulated after osteogenic induction, and SP1 overexpression further aggravated the effect of OM incubation. Osteogenic induction-increased ALP activity and mineralized nodules in BMSCs were abolished after the overexpression of SP1, which was partially alleviated by the downregulation of miR-133a-3p (Fig. 3E). Similarly, the levels of PINP, Colla1, OCN, and Runx2 were increased after osteogenic differentiation, which was inhibited by oe-SP1, whereas the miR-133a-3p inhibitor attenuated the effect of oe-SP1 under OM conditions (Fig. 3F-G). In total, SP1 aggravated osteogenic differentiation in BMSCs by modulating miR-133a-3p.

### MiR-133a-3p promotes osteogenic differentiation in BMSCs by targeting MAPK3

**Fig. 4:**
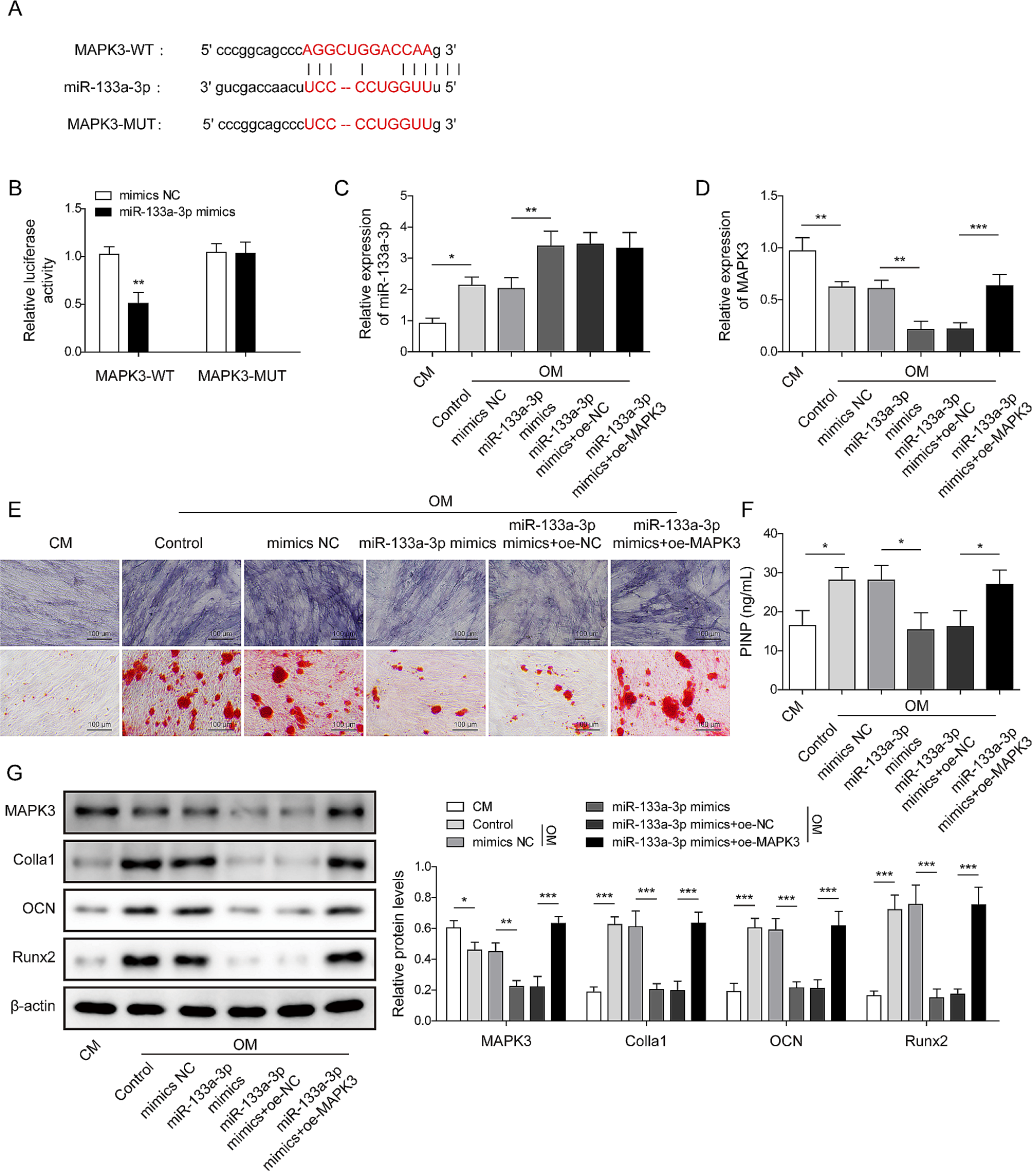
MiR-133a-3p promoted the osteogenic differentiation of BMSCs by targeting MAPK3. **A**, The binding site of miR-133a-3p and MAPK3 were predicted using Starbase. **B**, The binding between miR-133a-3p and MAPK3 was analyzed using dual-luciferase reporter assay. Subsequently, BMSCs were transfected with NC/miR-133a-3p mimics, miR-133a-3p mimics + oe-NC, or miR-133a-3p mimics + oe-MAPK3. **C**, The level of miR-133a-3p was examined using RT-qPCR. **D**, The expression of MAPK3 was examined using RT-qPCR. **E**, ALP activity was examined using Alkaline Phosphatase Kit, and the mineralized nodules in BMSCs were detected by Alizarin Red S staining (The scale bar was 100 μm). **F**, The content of PINP in BMSCs was assessed by commercial kits. **G**, The protein expression of MAPK3, Colla1, OCN, and Runx2 was assessed using Western blotting. *N* = 3, *p < 0.05, **p < 0.01, ***p < 0.001

The prediction of Targetscan (http://www.targetscan.org) and the investigation of dual-luciferase reporter assay indicated that MAPK3 was a downstream mRNA of miR-133a-3p (Fig. 4A–B). Next, BMSCs were transfected with miR-133a-3p mimics and/or oe-MAPK3 and then maintained in OM for 21 days. MiR-133a-3p mimics further aggravated OM-upregulated miR-133a-3p in BMSCs, whereas the overexpression of MAPK3 did not affect the level of miR-133a-3p (Fig. 4C). In contrast, the level of MAPK3 decreased after osteogenic induction, and miR-133a-3p overexpression further intensified the downregulation trend, whereas the effect was alleviated by the overexpression of MAPK3 (Fig. 4D). OM-upregulated ALP activity and mineralized nodules were alleviated by miR-133a-3p upregulation, which was greatly decreased by MAPK3 overexpression (Fig. 4E). Furthermore, miR-133a-3p mimics significantly downregulated the content of PINP in OM, the above result was reversed by oe-MAPK3 (Fig. 4F). Analogously, the downregulation of MAPK3 induced by osteogenic induction was intensified by miR-133a-3p mimics, whereas the overexpression of MAPK3 alleviated this phenomenon. Inversely, the OM-induced upregulation of Colla1, OCN, and Runx2 was attenuated by miR-133a-3p mimics, whereas the effect of miR-133a-3p mimics was reversed by MAPK3 overexpression (Fig. 4G). Overall, miR-133a-3p accelerated osteogenic differentiation in BMSCs by targeting MAPK3.

**Fig. 5:**
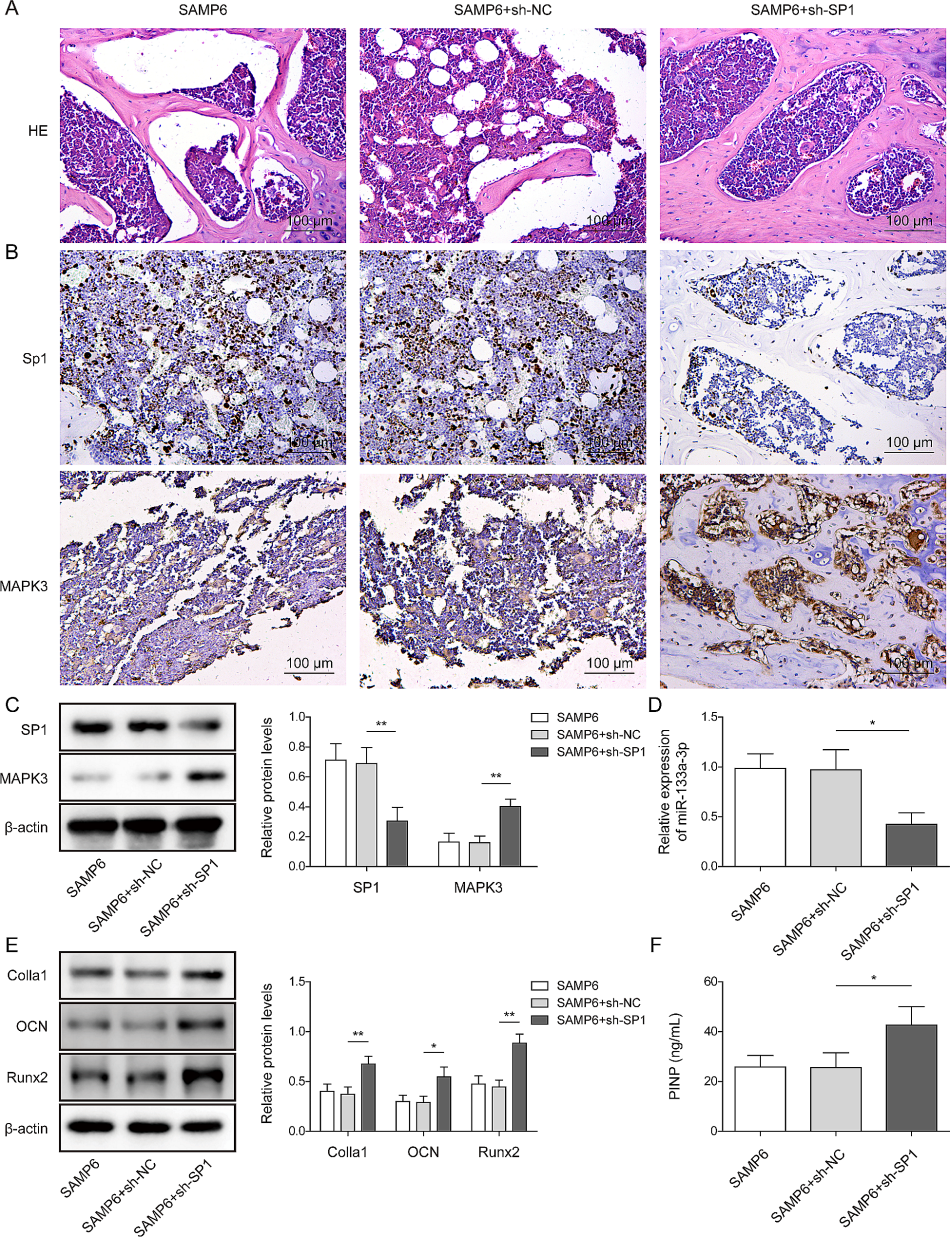
Knockdown of SP1 significantly alleviated the symptom of OPin vivo. (**A**) The histological changes in mice were observed using H&E staining. (**B**) The levels of MAPK3 and SP1 in mice were detected using IHC staining. (**C**) The protein levels of SP1 and MAPK3 in the tissues of mice were detected using Western blotting. (**D**) The level of miR-133a-3p in the tissues of mice was evaluated using RT-qPCR. (**E**) The protein levels of Colla1, OCN, and Runx2 in mice were examined using Western blotting. (**F**) The content of PINP in the serum of mice was measured using commercial kits. *N* = 6, *p < 0.05, **p < 0.01

### Knockdown of SP1 significantly alleviated OP progression in mice

To detect the function of SP1 in OP, knocking down SP1 in SAMP6 mice. As shown in Fig. 5A, loose trabeculae and reduced bone density in tissues of SAMP6 mice were significantly alleviated by SP1 knockdown. Furthermore, the expressions of SP1 in SAMP6 mice were significantly downregulated by SP1 knockdown. Adversely, MAPK3 upregulated in SP1 knockdown SAMP6 mice (Fig. 5B-C). Meanwhile, knockdown of SP1 could obviously decrease the level of miR-133a-3p, while increasing the levels of Colla1, OCN and Runx2 in SAMP6 mice (Fig. 5D-E). Furthermore, SP1 shRNA significantly inhibited the content of PINP in serum of mice (Fig. 5F). In summary, the knockdown of SP1 significantly alleviated OP progression in mice.

## Discussion

The pathogenesis of OP is induced by bone formation dysfunction [31]. Osteogenic differentiation in BMSCs is a complicated process that is affected by many factors [32]. Studies have indicated advances in OP treatment (e.g., siRNAs and lncRNAs) [33–35]. However, the outcomes remain limited. In this study, the data illustrated that SP1 inhibited osteogenic differentiation during OP progression in vitro and in vivo by mediating the miR-133a-3p/MAPK3 axis. This study elucidated the mechanism underlying the function of SP1 in OP.

BMSCs are a subtype of multifunctional differentiated cells that can differentiate into osteoblasts, osteocytes, and adipocytes, among others [36]. Furthermore, osteogenic differentiation in BMSCs is closely related to the occurrence of OP; the specific manifestation is that a decrease in osteogenic differentiation can induce bone formation disorders and promote OP progression [30]. Therefore, the osteogenic differentiation of BMSCs can alleviate the development of OP. This study explored the function of SP1 in osteogenic differentiation and identified a potential target for treating OP. The high expression of SP1 has been proven to be a risk factor for OP [37]. Our study indicated that SP1 was upregulated in the OP model. Mechanically, SP1 upregulated miR-133a-3p in vitro and in vivo. More importantly, this study confirmed that SP1 participates in osteogenic differentiation by mediating miR-133a-3p.

MiRNAs regulate the translation of mRNAs through target genes [38]. Furthermore, miRNAs can modulate the progression of various diseases, including OP [39]. Mao et al. found that miR-133a-3p regulates osteogenic differentiation by regulating the repeat domain of ankyrin [40]. MiR-133a-3p is also targeted by the lncRNA MEG3 to inhibit osteogenic differentiation during the progression of postmenopausal OP [29]. In these backgrounds, the expression of miR-133a-3p was decreased during the osteogenic differentiation of BMSCs. Similarly, this study proved that miR-133a-3p was upregulated in the OP model and that the miR-133a-3p inhibitor accelerated the osteogenic differentiation of BMSCs. These findings indicated that miR-133a-3p can attenuate the osteogenic differentiation of BMSCs. SP1 inhibited the osteogenic differentiation of BMSCs by transcriptionally regulating miR-133a-3p. Furthermore, MAPK3 was found to be directly targeted by miR-133a-3p, which can be activated by many factors. More importantly, MAPK3 participates in osteogenic differentiation. Mei et al. reported that resveratrol modulated cadmium-induced osteogenic differentiation through MAPK3 [41]. Furthermore, MAPK3 may also be involved in OP progression. Fang et al. demonstrated that artemisinin attenuated glucocorticoid-induced OP in BMSCs by inactivating the MAPK1/3 pathway [42]. Therefore, the aforementioned data illustrated that miR-133a-3p accelerated the osteogenic differentiation of BMSCs by targeting MAPK3. In summary, miR-133a-3p can prevent osteogenic differentiation by regulating MAPK3.

Indeed, this study has several limitations: more target mRNAs of miR-133a-3p in OP remain unexplored; the upstream mechanisms by which SP1 is upregulated in OP must be further investigated. Thus, it is important to investigate how SP1 interacts with miR-133a-3p. This will provide valuable insights into the molecular pathways involved in this disease. In summary, our research demonstrated that SP1 regulated the osteogenic differentiation of BMSCs by mediating the miR-133a-3p/MAPK3 axis. The mechanism underlying the function of SP1 in OP was elucidated. Our study may provide a novel theoretical basis for the treatment of OP.

### Electronic supplementary material

Below is the link to the electronic supplementary material.

Supplementary Material 1

## Data Availability

No datasets were generated or analysed during the current study.
