# Supplementary material for: SP1 regulates BMSC osteogenic differentiation through the miR-133a-3p/MAPK3 axis: SP1 regulates osteogenic differentiation of BMSCs
Source: J Orthop Surg Res. 2024 Jul 9;19:396. doi: 10.1186/s13018-024-04889-4 (PMC11232211; doi:10.1186/s13018-024-04889-4)
Supplement: Supplementary file 1 — Supplementary Material 1 [file 13018_2024_4889_MOESM1_ESM.pdf]

enago
Author First, Quality First
www.enago.cn

CERTIFICATE OF EDITING

This is to certify that the paper titled SP1 regulates BMSC osteogenic differentiation through the miR-133a-3p/MAPK3 axis has been edited for English language, grammar, punctuation, and spelling by Enago, the editing brand of Crimson Interactive Consulting Co., Ltd.

ISO 17100:2015
Translation Service Providers

ISO 27001:2013
Information Security Management System

ISO 9001:2015
Quality Management System

CRIMSON INTERACTIVE CONSULTING CO.LTD 克里门森互动(北京)顾问有限公司 1101030093948

Issued by:

Enago, Crimson Interactive (Beijing) Consulting Co., Ltd.Room 3217,
Cyber Tower A,
No. 2, Zhongguancun South Street,Haidian
District, Beijing

Disclaimer: The intent of the author's message has been preserved during the editing process. The author is free to accept or reject our changes in the document after reviewing our editing. This certificate has been awarded at the time of sharing the final edited version (full file or sections of the file) with the author. Enago does not bear any responsibility for any alterations done by the author to the edited document post **23rd Apr 2024.**

Japan	www.enago.jp, www.ulatus.jp, www.voxtab.jp	Russia	www.enago.ru
Taiwan	www.enago.tw, www.ulatus.tw	Arabic	www.enago.ae
China	www.enago.cn, www.ulatus.cn	Turkey	www.enago.com.tr
Brazil	www.enago.com.br, www.ulatus.com.br	S. Korea	www.enago.co.kr
Germany	www.enago.de	Global	www.enago.com, www.ulatus.com, www.voxtab.com

About Crimson:
Crimson Interactive Consulting Co. Ltd. is one of the world's leading academic research support services. Since 2005, we've supported over 2 million researchers in 125 countries with their publication goals.
